# Maternal Consumption of a Cafeteria Diet during Lactation Leads to Altered Diet-Induced Thermogenesis in Descendants after Exposure to a Western Diet in Adulthood

**DOI:** 10.3390/nu14091958

**Published:** 2022-05-07

**Authors:** Catalina Amadora Pomar, Catalina Picó, Andreu Palou, Juana Sánchez

**Affiliations:** 1 Laboratory of Molecular Biology, Nutrition and Biotechnology (Group of Nutrigenomics, Biomarkers and Risk Evaluation), University of the Balearic Islands, 07122 Palma, Spain; c.pomar@uib.es (C.A.P.); andreu.palou@uib.es (A.P.); joana.sanchez@uib.es (J.S.); 2 Health Research Institute of the Balearic Islands IdISBa, 07010 Palma, Spain; 3 CIBER de Fisiopatología de la Obesidad y Nutrición (CIBEROBN), 28029 Madrid, Spain

**Keywords:** diet-induced thermogenesis, metabolic programming, maternal diet

## Abstract

This study investigates the ability of a maternal cafeteria diet during lactation to program brown adipose tissue (BAT) metabolic responses to an obesogenic diet re-exposure in the adult offspring after consuming a standard diet (SD). Nursing rats were fed an SD or a cafeteria diet during lactation. Their offspring (O-C and O-CAF, respectively) were weaned onto an SD, and at 16 weeks of age they were switched to a Western diet until week 24. Gene and protein expression in BAT were measured at PN22 and at 24 weeks. At PN22, compared to controls, O-CAF rats displayed lower mRNA levels of lipogenesis-related genes (*Fasn*), and higher expression of genes related to lipolysis (*Pnpla2*), fatty acid uptake (*Cd36, Lpl*), and oxidation (*Cpt1b)*. Additionally, O-CAF animals displayed increased mRNA levels of *Adrb3*, *Ucp1*, and *Cidea*. In adulthood, these animals maintained lower mRNA levels of lipogenesis-related genes (*Pparg*, *Srebf1*, *Fasn*), but displayed lower expression of genes related to fatty acid uptake (*Cd36*), fatty acid oxidation (*Cpt1b*), lipolysis (*Pnpla2*), *Adrb3*, *Ucp1*, and *Cidea*. Thus, exposure to an obesogenic diet in nursing rats can affect long-term lipid metabolism and attenuate diet-induced thermogenesis in BAT in response to a new obesogenic dietary challenge later in life.

## 1. Introduction

Nutrition during early life can trigger adaptations that result in permanent changes in the physiology of the organism and produce long-term effects on the health status, a phenomenon known as “metabolic programming” [1,2]. In fact, increasing evidence from animal studies indicates that maternal obesity or a high-fat diet (HFD) consumption during gestation and lactation increases the susceptibility to developing metabolic syndrome-associated features in the offspring, including obesity, insulin resistance, and fatty liver disease [3,4,5,6]. Moreover, maternal overnutrition has also been described to exacerbate the negative outcomes of an HFD challenge later in life [7], but mechanisms involved remain poorly defined.

Thermogenesis takes place mainly in brown adipose tissue (BAT), and the main molecular effector of the process is the uncoupling protein 1 (UCP1) expressed in the inner mitochondrial membrane. UCP1 favours an excess of energy dissipation as heat allowing the free movement of protons across the mitochondrial membrane without ATP synthesis [8]. The adaptive thermogenesis refers to the dissipation of energy as heat in response to changing environmental conditions, for example, exposure to cold (cold-induced thermogenesis) or overfeeding (diet-induced thermogenesis) [9]. One particular animal model of diet-induced thermogenesis is the so-called “cafeteria diet”, in which rats fed a cafeteria diet exhibited a voluntary hyperphagia, but gained less weight than could be expected considering the energy content of the food ingested, which is associated with increased thermogenesis [10]. BAT mediates adaptive changes in metabolic rate by responding to the norepinephrine (NE), released from sympathetic terminals, through β-adrenergic receptors (AR), and in particular through the β3-AR [11,12]. As a consequence, it turns on a cascade of intracellular events that promotes UCP1 synthesis and activation, and brown adipocyte cell proliferation [13]. UCP1 activation could serve, in principle, to burn off the excessive caloric intake and maintain energy balance. In fact, experimental studies in rodents have shown that BAT activity protects against obesity [14,15]. Moreover, activated BAT takes up fatty acids and glucose from the circulation; therefore, their stimulation may help improve hypertriglyceridemia and hyperglycaemia [16,17,18]. Therefore, impaired diet-induced thermogenesis could contribute to diet-induced obesity and related metabolic complications, and an inadequate programming of BAT development may also contribute.

Gestation and lactation represent a sensible time window to BAT development, and nutritional and environmental factors during such periods may program long-term BAT function, as shown in rodents [19,20]. For instance, maternal undernutrition during gestation results in a diminished thermogenic capacity in BAT at early stages in the rat offspring, contributing, in part, to the higher propensity for fat accumulation and other metabolic alterations in adulthood [19]. In the same way, in mice, maternal HFD during lactation has been shown to impair adaptive thermogenesis in BAT at weaning and in adulthood in the male offspring [20], suggesting that the attenuation of BAT thermogenic function may be a key mechanism linking maternal overnutrition during lactation to long-term metabolic disorders in the offspring [20]. Moreover, diet normalization at weaning in mice exposed to maternal HFD during pregnancy and lactation was shown to be ineffective in completely reversing the altered thermogenic response to cold exposure in adulthood [21].

We previously described in rats that maternal intake of a cafeteria diet during lactation produces lasting effects on their offspring, characterized by an impaired glucose response, and greater fat content with no differences in body weight, similarly to the thin-outside–fat-inside phenotype [22]. At weaning, these animals displayed an altered response to fed/fasting conditions in the expression of key metabolic genes in liver and white adipose tissue (WAT), suggesting the existence of metabolic inflexibility already at this early age [23].

Therefore, considering the contribution of BAT function in long-term metabolic complications, the purpose of the present study was to evaluate whether maternal intake of a cafeteria diet during lactation also impaired BAT metabolism and thermogenesis capacity in male and female pups, both at weaning, in response to nutrition conditions during lactation, and in adulthood, when they were re-exposed to an obesogenic diet after an interval of a standard diet.

## 2. Materials and Methods

### 2.1. Ethics Statement

The animal protocol was reviewed and approved by the Bioethical Committee of the University of the Balearic Islands (Ref 3513 (26 March 2012)), following its guidelines for the use and care of laboratory animals.

### 2.2. Experimental Design

The animal protocol, regarding the treatment of dams, has been previously described in detail [22]. Virgin female Wistar rats housed at controlled temperature (22 °C), with a 12-h light–dark period and with free access to food and water, were mated with male rats. After mating, each dam was single caged. After delivery, each litter was adjusted to 10 pups per dam, and dams were randomly assigned to either control group or cafeteria group (*n* = 8 in each one). Control dams (C) were fed a standard chow diet (SD) and dams of the cafeteria group (CAF) were exposed to a cafeteria diet in addition to the SD. The cafeteria diet used was described in detail in [24].

**Figure 1:**
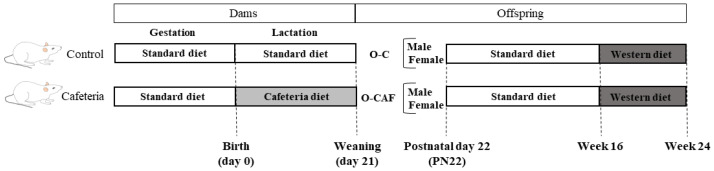
Experimental design scheme of the animal model used in this study. Abbreviations: PN22, postnatal day 22, O-C, offspring of control dams; O-CAF, offspring of cafeteria diet-fed dams.

On weaning, at postnatal day 22 (PN22), one set of male and female offspring of control and cafeteria dams (O-C and O-CAF, respectively) were killed under ad libitum feeding conditions by decapitation during the first 2 h of the light cycle. Another set of animals, siblings of the animals killed at PN22, were kept alive and were housed on a SD from weaning to the age of 16 weeks. After this period, the animals were exposed to a commercial obesogenic high-fat and high-sucrose diet (Western diet, WD; D12079B, de Research Diets) for 8 weeks. Two weeks before killing, saphenous vein blood samples were collected under 12-h fasting conditions. At the age of 24 weeks, O-C and O-CAF rats were killed by decapitation under ad libitum feeding conditions. At PN22 and at 24 weeks of age, interscapular BAT was rapidly removed and stored at −70 °C until analysis. Trunk blood samples were collected in heparinised tubes. Plasma was obtained from heparinised blood by centrifugation (1000× *g*, 10 min) and was stored at −20 °C until analysis. A scheme of the experimental design is shown in Figure 1.

Food intake and body weight were followed. Body fat content was measured at weaning, and at 16 and 24 weeks of age (by EchoMRI-700TM; Echo Medical Systems, LLC, Houston, TX, USA).

### 2.3. Analysis of Blood Parameters

An Accu-Chek Glucometer (Roche Diagnostics, Barcelona, Spain) was used to measure blood glucose concentration. Enzyme-linked immunosorbent assay kits were used for the quantification of leptin (R&D Systems, Minneapolis, MN, USA) and insulin (Mercodia AB, Uppsala, Sweden) in plasma samples. Enzymatic colorimetric kits were used for the quantification of plasma triglyceride (TG) levels (Sigma) and nonesterified (or free) fatty acids (Wako Chemicals GmbH, Neuss, Germany).

### 2.4. RNA Extraction and Gene Expression Analysis

Total RNA was extracted from interscapular BAT by using E.Z.N.A^®^ Total RNA Kit I (Omega Bio-Tek, Inc., Norcross, GA, USA) according to manufacturer’s instructions. Isolated RNA was quantified by using the NanoDrop ND-1000 spectrophotometer (NadroDrop Technologies, Wilmington, DE, USA), and its integrity was confirmed by using agarose gel electrophoresis.

Real-time quantitative polymerase chain reaction (RT-qPCR) was performed to measure mRNA expression levels of selected gene transcripts in BAT. Genes analysed were: Cd36 molecule (*Cd36*), lipoprotein lipase (*Lpl*), proliferator-activated receptor gamma (*Pparg*), stearoyl-coenzyme A desaturase 1 (*Srebf1*), fatty acid synthase (*Fasn*), patatin-like phospholipase domain containing 2 (*Pnpla2*), carnitine palmitoyltransferase 1B (*Cpt1b*), adrenoceptor beta 3 (*Adrb3*), uncoupling protein 1 (*Ucp1*), cell death-inducing DFFA-like effector a (Cidea), PPARG coactivator 1 alpha (*Ppargc1a*), PR/SET domain 16 (*Prdm16*), and T-box 15 (*Tbx15*). In short, total RNA (0.25 µg, in a final volume of 12.5 µL) was denatured at 65 °C for 10 min and then reverse transcribed to cDNA by using MuLV reverse transcriptase (Applied Biosystems, Madrid, Spain). RT-qPCR was performed by using the Applied Biosystems StepOnePlus Real-Time PCR Systems (Applied Biosystems, Madrid, Spain) according to the manufacturer’s instructions. Values for the threshold (Ct) were determined (StepOne Plus Software v2.3, Applied Biosystems, Madrid, Spain) and the relative gene expression was calculated as a percentage of the male control rats; guanosine diphosphate (GDP) dissociation inhibitor 1 (*Gdi1*) mRNA was used as reference gene.

### 2.5. UCP1 Protein Levels

A Western blot was performed to determine the protein levels of UCP1 in BAT of O-C and O-CAF animals at PN22 and at 24 weeks of age. Tissue samples were homogenized at 4 °C in 1:5 (*w:v*) in radioimmunoprecipitation assay (RIPA) buffer containing Halt Protease and Phosphatase Inhibitor Cocktail (Thermo Fisher, Rockford, IL, USA). A total of 10 µg of RIPA protein extracts were separated in a 4–20% SDS-PAGE (Criterion^TM^ TGX^TM^, Bio-Rad Laboratories, Madrid, Spain), and transferred to a nitrocellulose membrane (Bio-Rad Laboratories). After blocking, membranes were incubated with the primary antibody rabbit anti-UCP1 (GTX10983, GeneTex, Irvine, CA, USA), and HSP90 (4877, Cell Signaling, Danvers, MA, USA) was used as transfer and loading control. Afterward, membranes were incubated with the infrared-dyed secondary anti-IgG antibodies (Li-COR Biosciences, Lincoln, NE, USA). For infrared detection, membranes were scanned in Odyssey Infrared Imaging System (LI-COR Biosciences, Lincoln, NE, USA), and the bands were quantified by using the software Image Studio v5.2 (LI-COR Biosciences, Lincoln, NE, USA).

### 2.6. Statistical Analysis

No blinding was carried out for data analysis. All data are expressed as the mean ± standard error of the mean (*n* = 7–12). A Shapiro–Wilks normality test was used to check for normality. Levene’s test was performed to assess whether the variance was equal between groups for multi-group comparisons; data were log-transformed before analysis if the variance was heterogeneous. A two-way analysis of variance (ANOVA) was used to determine the effects of different factors: D, effect of maternal diet (control/cafeteria) during lactation, and S, effect of sex. Single comparisons were assessed with a Mann–Whitney test (*n* < 10 in some groups). All statistical analyses were executed in SPSS (SPSS, Chicago, IL, USA). The threshold of significance was defined at *p* < 0.05.

## 3. Results

### 3.1. Effects of Maternal Cafeteria Diet Intake during Lactation on Phenotypic Parameters and Food Intake in the Offspring

**Figure 2:**
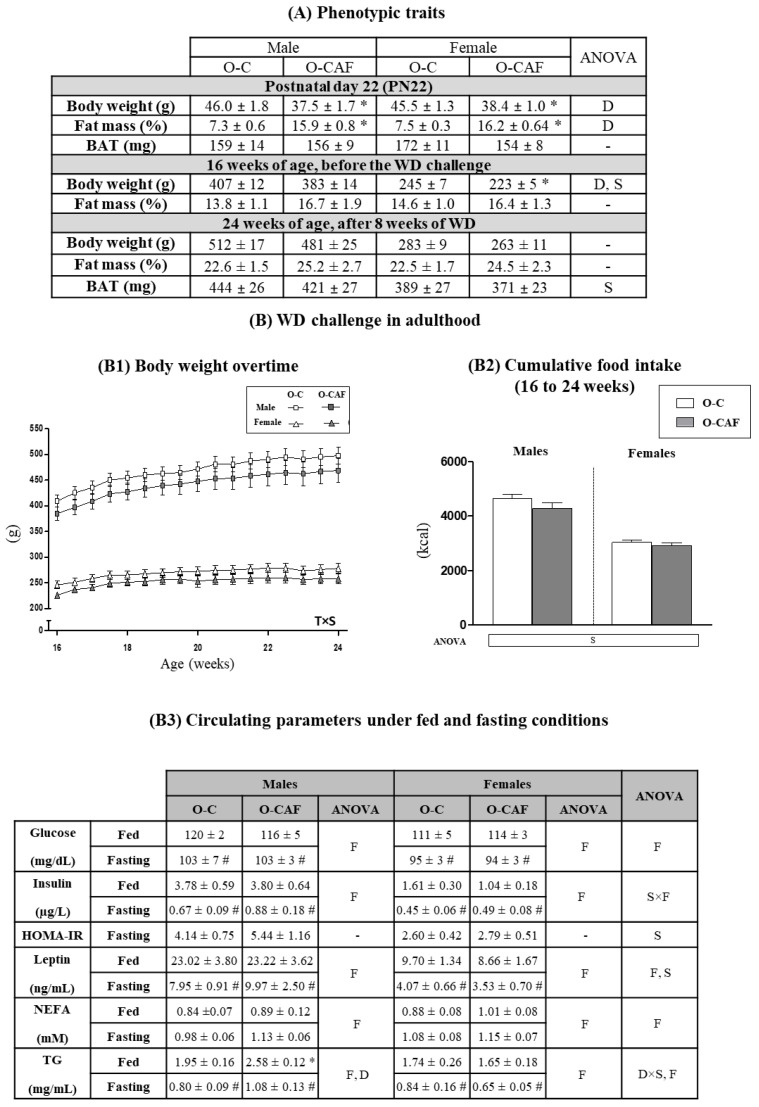
(**A**) Body weight, percentage of body fat, and weight of brown adipose tissue (BAT) in male and female offspring of control dams (O-C) and of cafeteria diet-fed dams during lactation (O-CAF) at postnatal day 22 (PN22), week 16 and week 24 of age. (**B**) Body weight overtime (**B1**), cumulative food intake (in kcal) (**B2**), and circulating parameters under fed and fasting feeding conditions (**B3**) of male and female O-C and O-CAF rats after the western diet (WD) challenge in adulthood. Data are expressed as the mean ± standard error of the mean of 7–12 animals per group. Statistics: D, effect of maternal diet (control/cafeteria) during lactation; S, effect of sex (male/female); F, effect of feeding condition (fed/fasting); D × S, interaction between maternal diet during lactation and sex; S × F, interaction between sex and feeding condition (*p* < 0.05, three and two-way ANOVA). T × S, interaction between sex and time, (*p* < 0.05, ANOVA repeated measures). *, O-CAF versus O-C (*p* < 0.05, Mann-Witney U test). #, Fasting versus feeding conditions (*p* < 0.05, Mann–Witney U test). Abbreviations: ANOVA, analysis of variance; HOMA-IR, homeostatic model assessment for insulin resistance; NEFA, nonesterified (or free) fatty acids; O-C, offspring of control dams; O-CAF, offspring of cafeteria diet-fed dams; TG, triacylglycerol.

Data of body weight and body composition at PN22 and until 24 weeks of age of animals from the same cohorts that were maintained throughout the whole period under a SD have been previously reported [22]. In this previous study, it was shown that despite dietary normalization after weaning, maternal intake of a cafeteria diet during lactation produced lasting effects in the metabolic health of their descendants, similarly to the thin-outside–fat-inside phenotype [22]. Here, we used another set of animals, from both O-C and O-CAF groups, siblings of the aforementioned animals, and they were exposed to WD from 16 to 24 weeks of age. Phenotypic parameters and food intake of animals are shown in Figure 2. As previously shown [22], at PN22, O-CAF animals presented lower body weight and greater fat mass percentage than O-C animals (Figure 2A). At 16 weeks of age, O-CAF rats maintained lower body weight (particularly females) than O-C rats, but no significant differences were found at 24 weeks, after an 8-week period of WD exposure. At 16 and 24 weeks, O-CAF rats also showed a trend to greater fat mass than O-C rats (*p* = 0.09, two-way ANOVA), but no significant differences were found between both groups. No differences were either observed in the weight of BAT between O-CAF and O-C animals, either at PN22 or in adulthood after 8 weeks of WD exposure. In addition, during the 8 weeks of WD exposure, no differences were observed in body weight overtime or in cumulative food intake between O-CAF and O-C animals, either males and females (Figure 2B1,B2). No differences were either found regarding body weight and body fat gain or in feed efficiency during the period of exposure to WD (data not shown). Regarding circulating parameters (Figure 2B3), at 24 weeks of age, there were no significant differences in most of the parameters analysed between O-CAF and O-C animals under ad libitum or fasting conditions. However, O-CAF males, but not females, showed a higher concentration of circulating TG than their controls, especially under feeding conditions.

### 3.2. Effects of Maternal Exposure to a Cafeteria Diet during Lactation on the Expression of Selected Genes in BAT of the Offspring

#### 3.2.1. Results in Young Animals (PN22)

**Figure 3:**
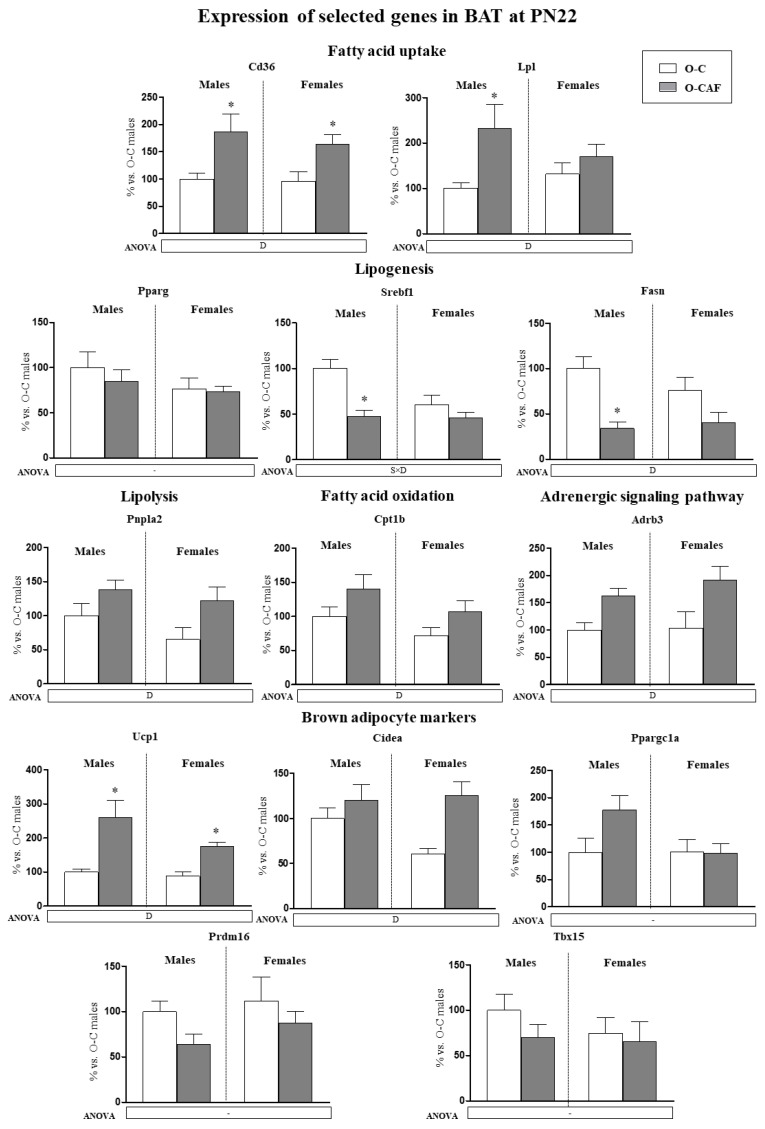
Expression of selected genes related with lipid metabolism (lipogenesis, lipolysis, fatty acid uptake, and fatty acid oxidation) and thermogenesis (adrenergic signalling and brown adipocyte markers) in brown adipose tissue (BAT) of male and female offspring of dams fed a control (O-C) or a cafeteria diet during lactation (O-CAF) ad libitum conditions at postnatal day 22 (PN22). mRNA levels were measured by real-time PCR and expressed as a percentage of the value of O-C males. The full names of the genes are indicated in the material and methods section. Data are expressed as the mean ± standard error of the mean of 6–8 animals per group. Statistics: D, effect of maternal diet (control/cafeteria) during lactation; S × D, interaction between sex and maternal diet (*p* < 0.05, two-way ANOVA). *, O-CAF versus O-C (*p* < 0.05, Mann–Witney U test).

The mRNA expression levels of selected genes related to lipid metabolism were studied in BAT of male and female offspring at PN22 (Figure 3). Maternal cafeteria diet consumption during lactation resulted in a decrease in mRNA levels of lipogenesis-related genes (*Srebf1* and *Fasn)*, especially in males, and in an increase in the expression of genes related to lipolysis (*Pnpla2)*, fatty acid uptake (*Cd36, Lpl*) and fatty acid oxidation (*Cpt1b*) in their offspring compared with control animals. Additionally, we analysed the expression of adrenoceptor beta 3 (*Adrb3*) and brown adipocyte marker genes in BAT (Figure 3). O-CAF animals, both males and females, displayed greater mRNA levels of *Adrb3*, *Ucp1*, and *Cidea* than O-C animals.

#### 3.2.2. Results in Adult Animals (24 Weeks) after Exposure to Western Diet

**Figure 4:**
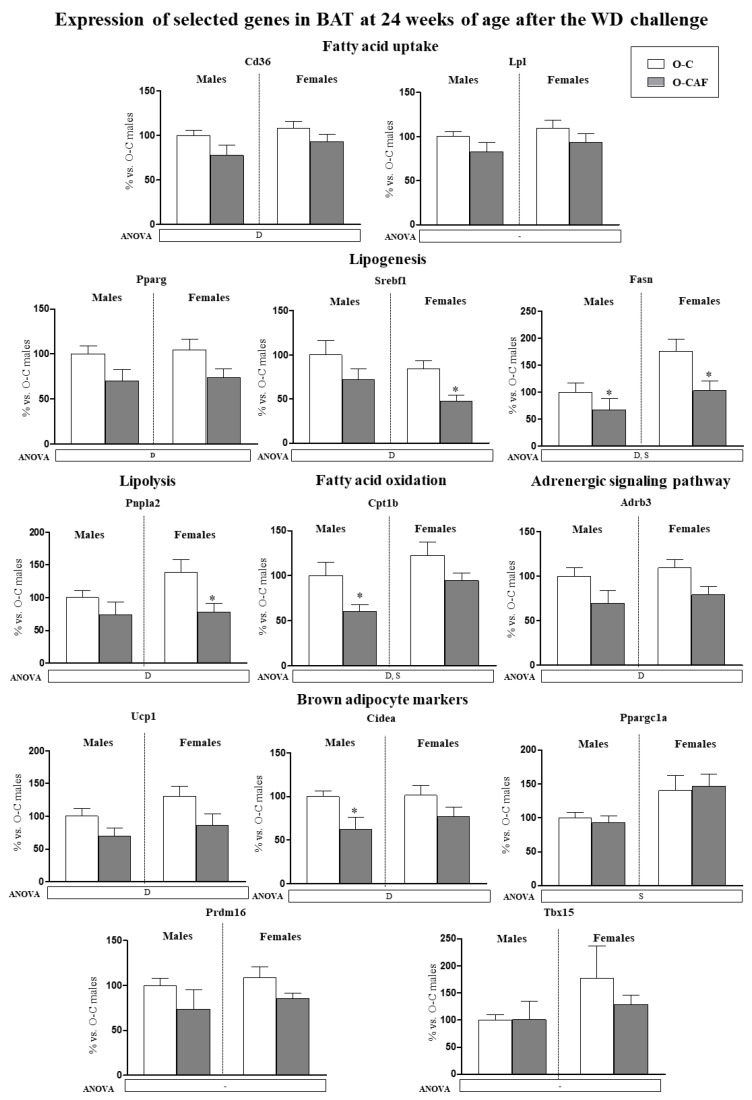
Expression of selected genes related with lipid metabolism (lipogenesis, lipolysis, fatty acid uptake, and fatty acid oxidation) and thermogenesis (adrenergic signalling and brown adipocyte markers) in brown adipose tissue (BAT) of male and female offspring of dams fed a control (O-C) or a cafeteria diet during lactation (O-CAF) at 24 weeks of age, after 8 weeks of a Western diet (WD). Animals were killed under ad libitum feeding conditions. mRNA levels were measured by real-time PCR and expressed as a percentage of the value of O-C males. The full names of the genes are indicated in the material and methods section. Data are expressed as the mean ± standard error of the mean of 8 animals per group. Statistics: D, effect of maternal diet (control/cafeteria) during lactation; S, effect of sex; (*p* < 0.05, two-way ANOVA). *, O-CAF versus O-C (*p* < 0.05, Mann–Witney U test).

Expression levels in BAT of the genes studied in young animals were also determined in 24-week-old animals, after 8 weeks of WD (Figure 4). At this age, a sexual dimorphism was observed in the expression of some of the genes analysed. Female animals displayed higher mRNA levels of *Fasn*, *Cpt1b* and *Ppargc1a* than males. In addition, O-CAF animals, compared to O-C, presented lower mRNA levels of genes related to lipogenesis (*Pparg*, *Srebf1*, and *Fasn*), lipolysis (*Pnpla2*), fatty acid uptake (*Cd36*), and oxidation (*Cpt1b*), as well as of *Adrb3*, *Ucp1*, and *Cidea* genes than controls.

#### 3.2.3. Results in Adult Animals (24 Weeks) That Were Maintained under Standard Diet

**Figure 5:**
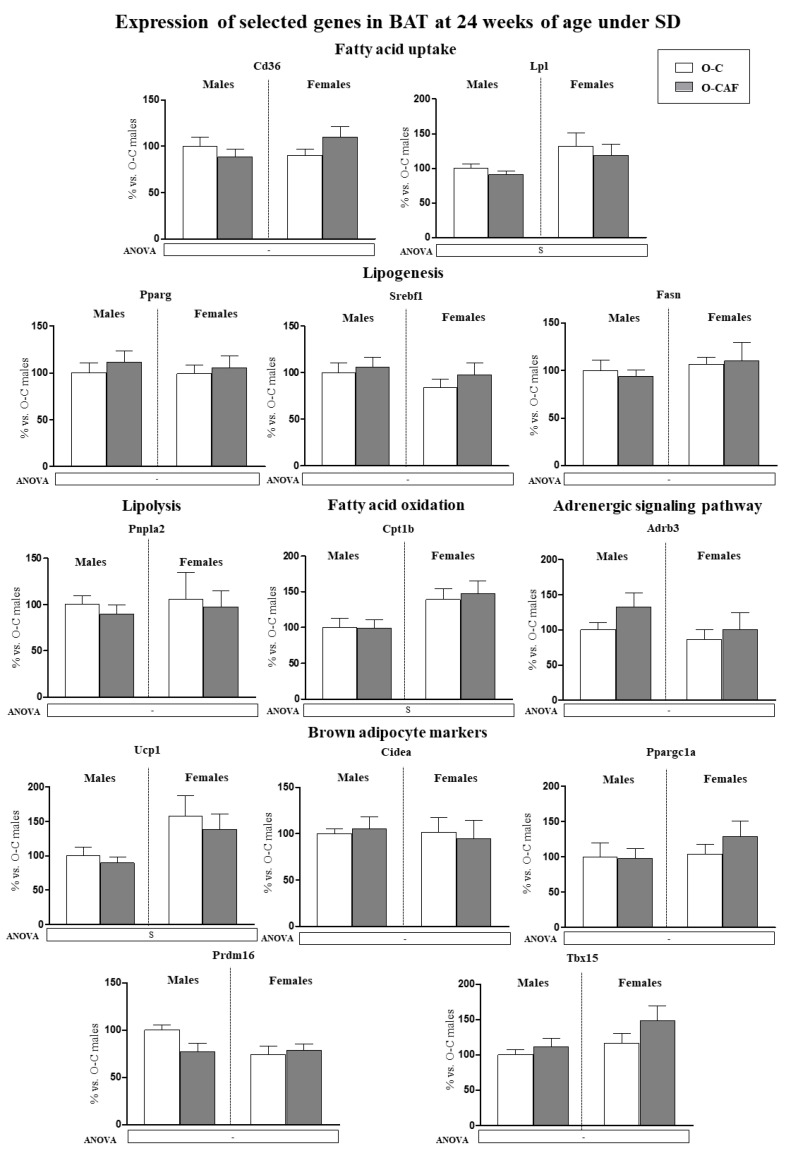
Expression of selected genes related with lipid metabolism (lipogenesis, lipolysis, fatty acid uptake, and fatty acid oxidation) and thermogenesis (adrenergic signalling and brown adipocyte markers) in brown adipose tissue (BAT) of male and female offspring of dams fed a control (O-C) or a cafeteria diet during lactation (O-CAF) at 24 weeks of age and maintained under a standard diet (SD) during the whole period. Animals were killed under ad libitum feeding conditions. mRNA levels were measured by real-time PCR and expressed as a percentage of the value of O-C males. The full names of the genes are indicated in the Materials and Methods section. Data are expressed as the mean ± standard error of the mean of 8 animals per group. Statistics: S, effect of sex; (*p* < 0.05, two-way ANOVA).

To find out if the differences observed in the gene expression profile in BAT between O-CAF and O-C animals after the 8-week period of WD exposure are indeed attributable to a different response to WD, we analysed the expression of the aforementioned genes in animals from the same cohorts (both O-C and O-CAF) that were maintained from weaning to 24 weeks of age under SD (Figure 5). Phenotypic features of these animals have been previously described [22]. Interestingly, no significant differences were observed in the expression levels of the genes studied between O-CAF and O-C animals that were maintained on a SD. We found sex-associated differences, because female animals displayed higher mRNA levels of *Lpl*, *Cpt1b*, and *Ucp1* than males.

### 3.3. Western Blot Analysis of UCP1 in BAT in Young Animals (PN22) and in Adult Animals (24 Weeks) after Exposure to Western Diet

**Figure 6:**
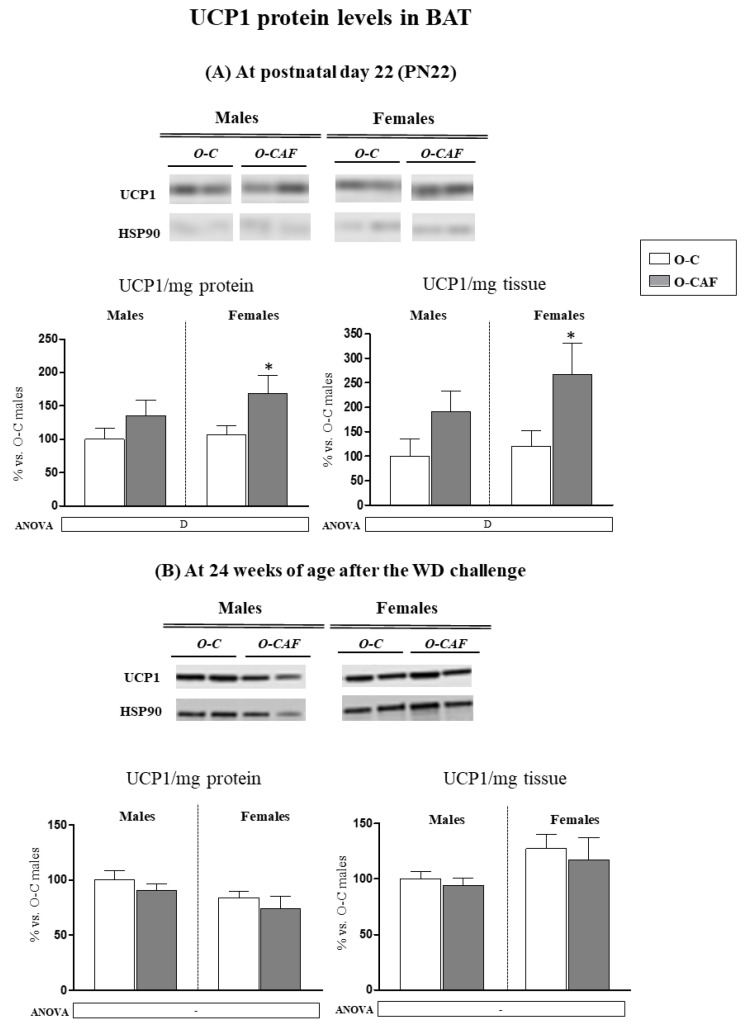
Specific levels of UCP1 (UCP1/mg protein) and of UCP1/mg of tissue in brown adipose tissue (BAT) of male and female offspring of dams fed a control (O-C) or a cafeteria diet during lactation (O-CAF) at postnatal day 22 (**A**) and at 24 weeks after 8 weeks of a Western diet (WD) (**B**). Data are expressed as the mean ± standard error of the mean of 6–12 animals per group and expressed as a percentage of the value of O-C males. Representative bands of UCP1 and HSP90 (reference protein) for each animal group are shown. Statistics: D, effect of maternal diet (control/cafeteria) during lactation (*p* < 0.05, two-way ANOVA). *, O-CAF versus O-C (*p* < 0.05, Mann–Witney U test).

Protein levels of UCP1 were also determined in BAT at PN22 and in adulthood, at 24 weeks of age after 8 weeks of exposure to WD. At PN22, O-CAF animals showed greater levels of UCP1, both when expressed per mg tissue protein (specific UCP1) and per mg tissue, compared with O-C (Figure 6A). No differences were observed in adulthood (Figure 6B).

## 4. Discussion

Maintenance of health status requires the capacity to continuously adapt to the changing environmental conditions by metabolic response reactions to maintain homeostasis [25]. Perinatal nutrition could program the response to a nutritional challenge later in life. For example, it has been described that the offspring of mice exposed to a HFD during the perinatal period suffered a more deleterious response to a second exposure to an HFD in adulthood [7]. Here we show that the offspring of cafeteria diet-fed dams during lactation displayed, at weaning, early adaptations in the expression profile of genes related to lipid metabolism and thermogenesis in the BAT that would be aimed at counteracting the higher caloric intake from maternal milk. However, in adulthood, and after a WD challenge, these animals showed a lack of response to this new obesogenic stimulus, suggesting that the thermogenic capacity in BAT was impaired.

BAT develops and differentiates during fetal life, because newborns require the presence of BAT to survive (to protect against cold) [26]. Nutritional environment during gestation and lactation could affect long-term BAT function [19,20]. This is of relevance because inadequate diet-induced thermogenesis has been shown to contribute to diet-induced obesity [15]. The thermogenesis in BAT is mainly activated via β3-adrenergic receptors [11]. In rats, mechanisms of β-adrenergic modulation of gene expression in brown fat are already established at birth [27]. It is known that the sympathetic nervous system (SNS) is activated upon feeding and contributes to diet-induced thermogenesis in BAT [28,29]. Concretely, hyperlipidic diets are known to increase *Ucp1* expression in BAT [30]. Here we show that on PN22, just one day after weaning, the offspring of dams fed a cafeteria diet during lactation presented greater mRNA and protein expression levels of *Ucp1* in BAT compared to their controls. This was accompanied with the presence of increased mRNA levels of *Cidea*. No differences were observed in the expression of *Prdm16* between O-CAF and O-C animals. Although *Prdm16* has been considered a key regulator of brown adipocyte differentiation, changes in its mRNA expression are not always linked to those of *Ucp1* [31,32]. O-CAF animals also presented, at weaning, greater mRNA levels of *Adrb3,* which encodes the predominant regulator of BAT thermogenesis in rodents (ADRB3) [11], and genes related with lipolysis (*Pnpla2*), fatty acid uptake (*Cd36*, *Lpl*) and oxidation (*Cpt1*), and decreased expression of lipogenesis-related genes (*Srebf1*, *Fasn*) in BAT than controls. This suggests a higher sympathetic activation and hence a higher lipolysis of their local TG, to keep a free fatty acid (FFA) supply for the increased thermogenesis. In addition, FFA can be obtained from the circulation by the action of lipoprotein lipase, which catalyses the release of fatty acids from TG included in the lipoproteins, and by the action of CD36, which transports FFA into the cell [16]. Later, FFA will then be channelled to the mitochondria, with the participation of carnitine palmitoyltransferase 1, and they will not only serve as energy substrates but also will activate UCP1 [33,34]. Thus, offspring of dams fed a cafeteria diet during lactation presented a gene expression profile in the BAT consistent with an activated diet-induced thermogenesis, probably to face the higher fat content of maternal milk. In turn, the reduced expression of genes related to lipogenesis in young O-CAF rats is probably reflecting the higher fat content of their diet [35]. In fact, we have previously shown that milk from cafeteria diet-fed dams during lactation had a higher lipid content and a higher percentage of energy from lipids than milk from control rats [22]

Therefore, the activated BAT thermogenesis in O-CAF pups found at weaning may be related with the greater lipid overload provided by milk during the suckling period because of maternal diet [22]. However, besides the amount of fat, the type of fat may differently affect diet-induced thermogenesis [36]. We have previously described that the offspring of olive oil-supplemented rats during lactation showed higher levels of UCP1 in BAT at weaning, in comparison with the offspring of dams supplemented with butter or margarine, probably due to the higher oleic acid content in milk [36]. Interestingly, milk of cafeteria-fed dams during lactation has been shown to be enriched in long-chain FA and contain greater content of oleic acid (C18:1) than control dams [37], and this could contribute to the increased thermogenesis capacity in O-CAF rats. Therefore, at early age, O-CAF animals attempt to dissipate excess energy intake supplied by the milk and/or by the direct intake of the cafeteria diet at the end of the lactation period. However, the activation of diet-induced thermogenesis observed in these animals was not enough to avoid excess fat accretion and to counteract the detrimental effects of the maternal obesogenic diet observed in these animals [22,23].

A different picture was observed in adult O-CAF animals after a second obesogenic dietary challenge, because they showed an impaired response to the diet. Specifically, adult O-CAF animals, both males and females, presented an altered expression profile of genes related to lipid metabolism in BAT, and a disrupted thermogenic response to the obesogenic diet insult, in comparison to O-C animals. A lower activation of the thermogenesis in adult O-CAF animals is evident because of the presence of reduced expression levels of *Ucp1* and *Cidea* compared to control animals, with no changes in the UCP1 protein levels. Adult O-CAF animals also presented lower mRNA levels of genes related to lipolysis (*Pnpla2*), fatty acid uptake (*Cd36*) and oxidation (*Cpt1b*), and lipogenesis (*Pparg*, *Srebf1* and *Fasn*) in BAT than their controls. This suggests a reduced fatty acid supply and thermogenesis activation in BAT to face the WD, that may be related, at least in part, to a reduced expression of the *Adrb3* gene. Permanent changes in BAT thermogenesis through the sympathetic nervous system-mediated alterations have been demonstrated by early postnatal overnutrition (with a reduction of the litter size per dams) [38]. In adulthood, these animals displayed a reduced thermogenic capacity, lower BAT *Ucp1* expression and reduced responsiveness to cold [38]. In addition, in mice exposed to HFD during pregnancy and lactation, diet normalization after weaning failed to completely reverse the ability to activate the thermogenic program after cold exposure, and this was related to a lower sympathetic activation [21]. Other studies have also shown that maternal HFD during lactation has lasting effects on BAT function in offspring [20]. At weaning, offspring of HFD-fed dams had higher *Ucp1* expression than controls; however, these animals showed impaired thermogenic adaption under cold stimulus [20]. At 16 weeks of age, and similar to our results at 24 weeks of age, offspring of HFD-fed dams had lower *Ucp1* and *Cidea* expression in BAT than offspring of control dams [20]. The impairment of BAT thermogenesis was partially due to the attenuation of cellular β3-adrenergic signalling [20]. In fact, decreased levels of β-adrenergic receptors have been associated with obesity. Concretely, mice with triple knockout for β-adrenergic receptors (*Adrb1*, *-2*, and *-3*) are severely obese when fed a high-energy diet and exhibited lower UCP1 levels [39]. Conversely, animals treated with ADRB3 agonists exhibit greater BAT activation and *Ucp1* expression [40,41,42] and increased lipid mobilization [40,41].

It is noteworthy that the differences in the gene expression profile in BAT between O-CAF and O-C rats, when exposed to a WD diet in adulthood, have not been observed between animals from the same cohorts (O-CAF and O-C) that were maintained under an SD throughout the period. This supports the hypothesis that the offspring of dams exposed to a cafeteria diet during lactation have an altered thermogenic response to a new obesogenic insult in adulthood; that is, they have lost the metabolic flexibility that they had at an early age and that control animals do maintain in adulthood. This lack of response could lead to metabolic alterations over time, particularly if animals are exposed to obesogenic environments later in life. However, it must be highlighted that, in the present study, despite the thermogenic response is apparently impaired in adult O-CAF rats, phenotypic traits (i.e., body weight and fat mass percentage, and most circulating parameters analysed) in these animals after two months of WD feeding were not different than controls. One exception is circulating TG, which were higher than controls, but only in the male offspring. We cannot rule out that this situation may worsen over time, because this period of WD exposure may not have been sufficient to make a clear difference with respect to their controls, which were also exposed to this obesogenic diet. On the other hand, the fact that the metabolic alterations are more evident in males than females seems to be consistent with the fact that they generally show a worse response to an obesogenic diet, with a higher tendency to suffer metabolic-syndrome related alterations [43].

In conclusion, present results suggest that the diet-induced thermogenic response in BAT in adulthood may be influenced by maternal nutrition during lactation. At early ages, rat pups are responsive to maternal exposure to cafeteria diet during lactation, displaying an increased mRNA expression of thermogenesis-related genes in BAT, including *Adrb3*, genes related to lipolysis, fatty acid uptake and oxidation, and *Ucp1* and *Cidea*, to attenuate the high lipid overload supplied by milk. However, such nutritional conditions during the suckling period seems to alter the thermogenic response in adulthood after exposure to a second dietary challenge. Therefore, lactation seems to be a critical time-window for BAT thermogenic function programming, so that alterations during this period could increase the susceptibility to develop metabolic-related dysfunctions in adulthood. These results provide further evidence of the importance of adequate maternal nutrition during lactation as a good strategy to prevent metabolic disorders in the adult offspring.

Since its rediscovery in adult humans [44], BAT has been in the spotlight of many human studies, and its activation capacity has emerged as a promising therapeutic tool against obesity and related pathologies. Therefore, if these results could be extrapolated to humans, they would provide new insights for practical nutritional recommendations in lactating mothers in relation to the programming of BAT activity in infants, which could allow them to better cope with eventual overfeeding later in life and improve overall metabolic health. Thus, improving nutritional conditions during early life could represent a possible approach to facilitate a better BAT response in adulthood and attenuate the high prevalence of metabolic diseases.
